# Proteome dynamics during establishment of California grunion (*Leuresthes tenuis*) cell lines

**DOI:** 10.1186/s12915-026-02577-9

**Published:** 2026-03-25

**Authors:** Meranda M. Corona, Johnathon Li, Dietmar Kültz

**Affiliations:** Department of Animal Science and Genome Center, University of California, Davis, Davis, CA USA

**Keywords:** DIA proteomics, Immortalization, Senescence, Cell line establishment, RNA processing, Proteostasis, Adhesion/ECM, Fish cell line, Transition window

## Abstract

**Background:**

Establishing continuous cell lines is hindered by limited molecular resolution of culture establishment and the transition to sustained proliferation. Here, proteome changes across passages were quantified in two independently derived California grunion (*Leuresthes tenuis*) embryonic-derived cell cultures, alongside morphology and growth metrics, to identify proteome dynamics associated with early establishment and subsequent stabilization of continuous proliferation under the standard conditions.

**Results:**

Morphological analysis identified a reproducible transition window centered on passage 4 (P4), coincident with changes in growth trajectories and consolidation toward epithelial-like morphology in three replicate cell lines LtE-1, LtE-2, and LtE3. Two of these replicate lines (LtE1 and LtE2) were analyzed by quantitative cell population proteomics confirming this transition, consistent with establishment-associated selection and/or cell-state change in a mixed early culture. Marker proteins associated with epithelial identity increased in abundance while fibroblast-associated markers declined. Across the transition window and subsequent passages, LtE-1 and LtE-2 shared broad remodeling of biosynthetic, proteostatic, adhesion/ECM, and lipid-related functions, and recurrence-filtered interaction networks highlighted passage-linked module consolidation. However, LtE-1 and LtE-2 differed in their temporal trajectories (transient surges in LtE-1 versus sustained reinforcement in LtE-2). Because early cultures contained mixed morphologies and cell population proteomics integrates across subpopulations, these patterns are presented as proteome dynamics of establishment and candidate biosignatures rather than definitive cell-intrinsic mechanisms.

**Conclusions:**

Passage-resolved cell population proteomics in two replicate California grunion embryonic-derived cell cultures define a baseline of establishment-associated remodeling and identifies candidate biosignatures linked to a reproducible transition window and subsequent stabilization of proliferation. The resulting passage-resolved baseline motivates lineage-resolved validation to distinguish the relative contributions of selection, cell-state transitions, and media adaptation to the observed establishment trajectory.

**Supplementary Information:**

The online version contains supplementary material available at 10.1186/s12915-026-02577-9.

## Background

Immortalized cell lines, or cells which continuously replicate in vitro, are defined by their capacity to proliferate indefinitely under ideal culture conditions [1]. Essential tools for physiology, molecular biology, and toxicology, these cultures serve as a consistent and renewable alternative to primary cells and reduce the need for whole-animal experimentation [2]. Once established, immortalized cell lines are especially well-suited for high-throughput workflows, genetic manipulation, long-term experimental design, and reproducibility across laboratories. This is especially true for non-model systems, as they also expand access to species that are seasonally or logistically restricted. Corresponding cell lines can be stored and shared without requiring additional animal sacrifice. Immortalized cell lines also embody the principles of minimizing in vivo research, as they are considered a more ethical option in experimentation at the cellular and lower levels of biological organization [3–6].

In contrast to immortalized cell lines, primary cultures are constrained by a finite lifespan. Many will undergo senescence within weeks to months, generally due to factors such as mitochondrial dysfunction, accumulation of DNA damage, or cell cycle arrest [7–11]. While senescence can sometimes be bypassed through genetic modification or pharmaceutical intervention, such as the introduction of viral oncogenes or the use of RHO-associated kinase inhibitors, these approaches are often complex, technically demanding, and not universally successful [12, 13]. In many non-traditional models, the establishment of continuously replicating cell lines can remain a challenge even with such interventions [2]. Yet, fish cells in culture have long stood out by their ability to spontaneously immortalize into continuously replicating cell lines [14, 15]. Establishment of these fish cell lines are often documented using morphology and propagation metrics, but the molecular programs that coincide with early passage transitions are rarely quantified at high resolution across passages. This, along with the stochastic success of existing approaches, highlights the need to better understand the intrinsic molecular pathways that characterize the transition of cell lines from finite to continuous growth in vitro [16]. The identification of early molecular signatures associated with continuously replicating cell line establishment may provide new molecular targets for advancing cell line development that are more reliable than traditional trial-and-error methods [17].

An emerging model system for such investigations is the California grunion (*Leuresthes tenuis*), a coastal marine silverside fish. Unlike many vertebrate species, cell cultures derived from *L. tenuis* embryos can be maintained through extended passaging and exhibit only brief interruptions in proliferative capacity during early establishment without the need for exogenous treatment. Preliminary reports have found that cell cultures derived from this species rapidly progress to sustained proliferation and can be maintained for extended passages [18, 19]. Based on this observation, a hypothesis was formulated that California grunion cell cultures reproducibly engage endogenous programs during early establishment that coincide with stabilization of proliferation and predictable remodeling of quantitative proteome profiles, without pharmacological or genetic intervention. Repeated establishment of long-term proliferative cultures across independently derived cell lines makes *L. tenuis* a compelling model for exploring how immortalization may arise in vitro [20, 21]. Additionally, as California grunion cannot be bred in captivity and eggs and embryos are only available during a brief annual spawning window in nature, robust cell lines also serve as practical tools for cellular and molecular biology studies of this species year-round [22–26].

In this study, three independently derived long-term proliferative cell lines (LtE-1, LtE-2, and LtE-3) were established from California grunion embryonic-derived tissue. Quantitative proteomics was used to characterize two of these lines (LtE-1 and LtE-2) and to characterize proteome dynamics associated with establishment and acquisition of sustained proliferation in vitro. LtE-1 and LtE-2 were compared across multiple passages starting as early as the first passage of primary culture. The cell proteomes were quantified to identify shared and divergent functional profiles that were enriched at specific passage numbers. Moreover, proteomic signatures were assessed to identify biochemical pathways differentially regulated during the transition to continuous growth in vitro. LtE-1 was sampled across passages (P) 1–12 and LtE-2 across P4-P7, providing both a longitudinal baseline and an early window of culture progression.

This study tested the hypothesis that acquisition of sustained proliferation during early establishment is accompanied by both marked transient adjustments and sustained proteomic changes that reconfigure growth-support programs and growth-limiting restraints at specific early passages. To test this hypothesis, the functional enrichment analyses, temporal trendlines, and protein subnetworks were combined with quantitative proteomics data for early passages spanning the transition from primary culture to sustained proliferation in vitro. This approach enabled tracking of shared and divergent pathways across independently established continuously proliferating cell lines [27].

## Results

**Fig. 1:**
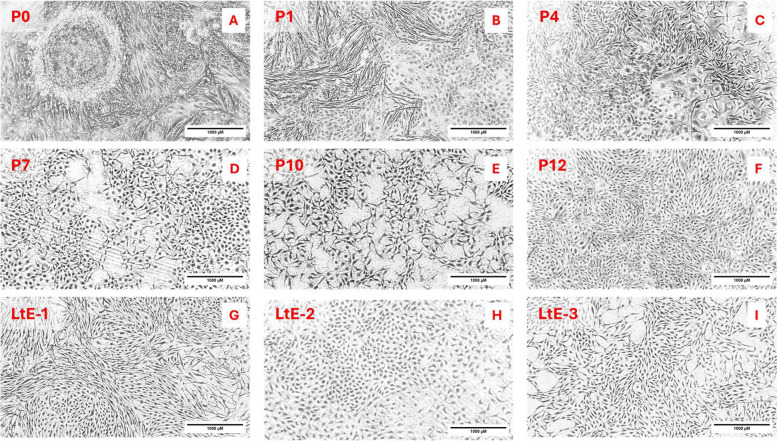
**A-G**: Morphological progression of the LtE-1 cell line across passages. Phase-contrast microscope photographs (10x) depict the LtE-1 cell line at key stages during the immortalization process: **A** passage 0 (primary explant stage), **B** passage 1 (imaged approx. 2 weeks after passage), **C** passage 4, **D** passage 7, **E** passage 10, and (**F**) passage 12. Two weeks after initiation of the culture, proliferative outgrowth was observed from the tissue fragments (**A**-**B**). Early passages displayed a heterogeneous morphology, consisting of both fibroblast-like and epithelial-like cells. By passage 4 (**C**) a cuboidal, epithelial-like morphology was dominant and remained consistent in subsequent passages (**D**-**F**). **G**-**I**: Stable morphology at extended passages for the three independently-derived cell lines. **G** LtE-1 (at P33), **H** LtE-2 (P10), **I** LtE-3 (P35). The late-passage images show that grunion embryonic-derived cells reproducibly achieve continuous proliferation across three independent derivations while maintaining stable morphology. Passage number and line identity are indicated by text overlays in the upper-left corner of each panel. Scale Bar: 1000 µm

To characterize passage-resolved proteome dynamics during cell line establishment, the proteomes of two independently derived cell lines were analyzed across sampled passages. LtE-1 was sampled from passage (P) 1 to P10 and P12, providing early- and late-passage context. LtE-2 was sampled from P4 to P7, enabling direct crossline comparison across the transition window and early post-transition passages (the period encompassing the transient slowing of proliferation observed in t80/PD trajectories). Representative higher-passage number images for LtE-1, LtE-2, and LtE-3 are included to illustrate stable morphology at extended passages across three independent derivations (Fig. 1).

### Morphological progression establishes passage-level context

Phase-contrast microscopy of LtE-1 cultures revealed progression from early heterogeneity to later uniformity of cell type (Fig. 1A-G). Early passages displayed mixed cell morphologies, varying between rounded and elongated phenotypes. By passage 4 (Fig. 1C), there was a shift into a stabilized morphology. Fibroblast-like cells diminished, and a more uniform cuboidal, epithelial-like morphology became dominant, coinciding with the transient slowing and subsequent resumption of faster proliferation inferred from t80/PD trajectories. Persistent morphological changes establish passage 4 as a practical baseline for interpreting proteomic changes across the transition window sampled here. Late-passage morphology for all three independently derived cell lines is shown in Fig. 1(G-I). At confluency under routine conditions, each line retains uniform epithelial-like cells with consistent shape. The images extend the early-mid passage series, indicating that stabilization of morphology after passage 4 persists at extended passages.

### Proliferation kinetics across passages

**Fig. 2:**
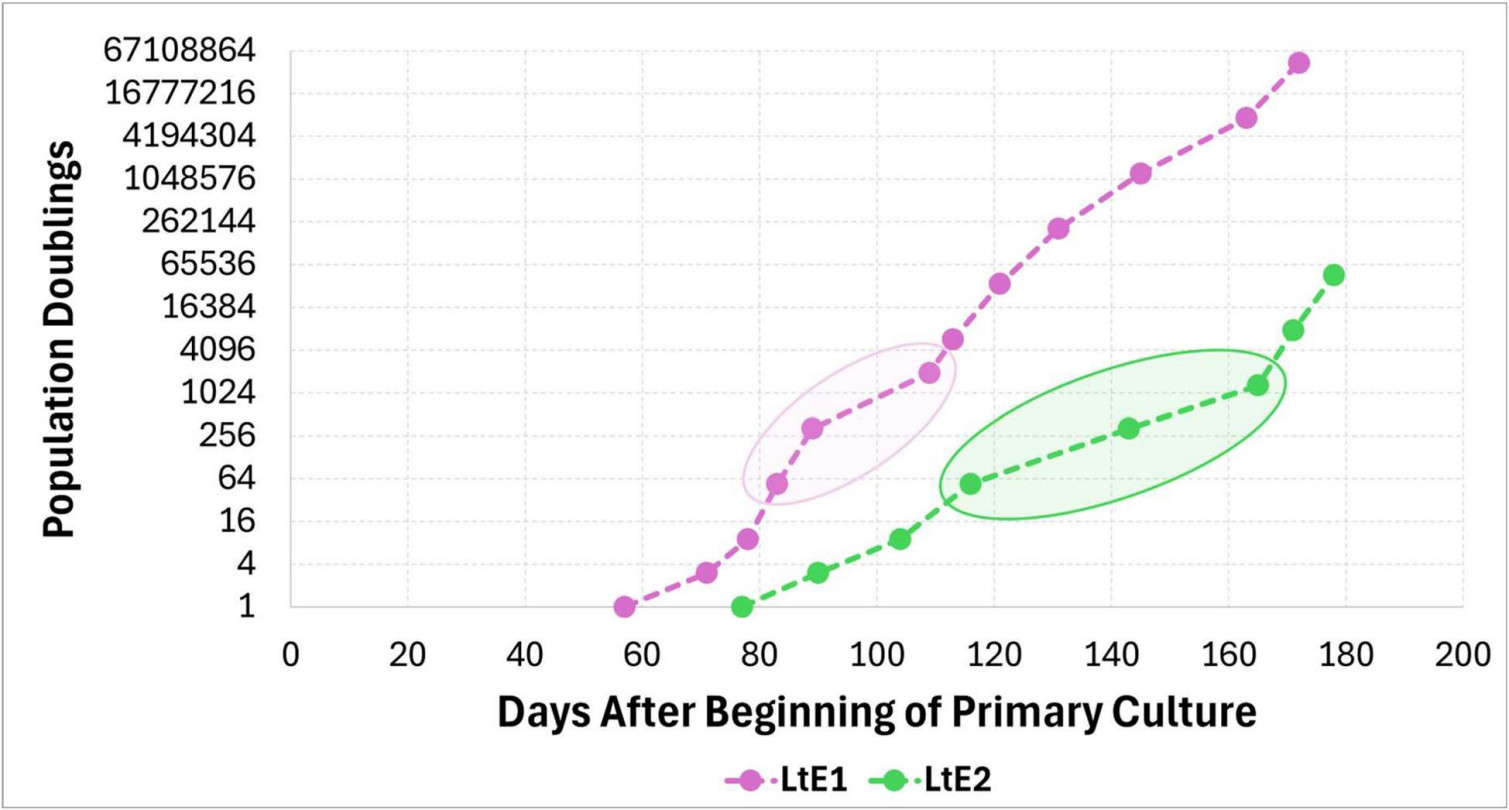
Proliferation kinetics across passages. Cumulative population-doubling (PD) series from passage 1 (P1), plotted against days after beginning of primary culture. Each point indicates a passage. Both LtE-1 and LtE-2 lines show a reduction in proliferative capacity attributed to a transient reduction in proliferative capacity between P4 and P6, followed by accelerated proliferation at later passages. Ellipses mark the transient reduction in proliferative capacity (LtE-1, 83–109 d; LtE-2, 116–165 d). Pre-P1 outgrowth is not included in PD counts

Population growth was tracked as time to ~ 80% confluency (t80) and as a cumulative population-doubling (PD) series recorded from P1 (values in Additional file S1; curves in Fig. 2). In LtE-1, t80 shortened from 57 days (d) at P1 to 14 d at P2 and 7 d at P3, remained low at 5–6 d at P4-P5, then increased transiently at P6 (20 d), and then was reduced again to 4–10 d by P7-P12. In LtE-2, t80 was 77 d at P1, 13–14 d at P2-P3, 12 d at P4, peaked at 22–27 d at P5-P6, and shortened to 6–7 d at P7-P8. The local t80 maxima occurred when the recorded population-doubling series reached 1,944 at P6 in LtE-1 and 324–1,296 at P5-P6 in LtE-2. Together, these measurements show a brief slowing of proliferation during P4-P6 (inferred from t80/PD trajectories), followed by resumption of faster proliferation at later passages for both LtE-1 and LtE-2. Here, stages are defined operationally by passage number: P1–P3 represent early establishment with mixed morphology, P4 is treated as the pivot passage, P4–P6 comprise the transient slowdown (transition window), and P7 + denote post-transition passages with sustained growth under our culture conditions.

Operationally, P4 was treated as the pivot because it marks the first passage after which both growth trajectories and proteome profiles change direction relative to earlier passages. In LtE-1, this window is bracketed by a transient elevation in t80 through P5-P6 followed by shortening of t80 at later passages, while LtE-2 shows a similar but slightly shifted pattern. The proteomics analyses were therefore structured around comparisons to P4 to test whether coordinated remodeling aligns with this reproducible transition window rather than changing gradually across passages.

### Epithelial and fibroblast markers across passages in LtE-1

**Fig. 3:**
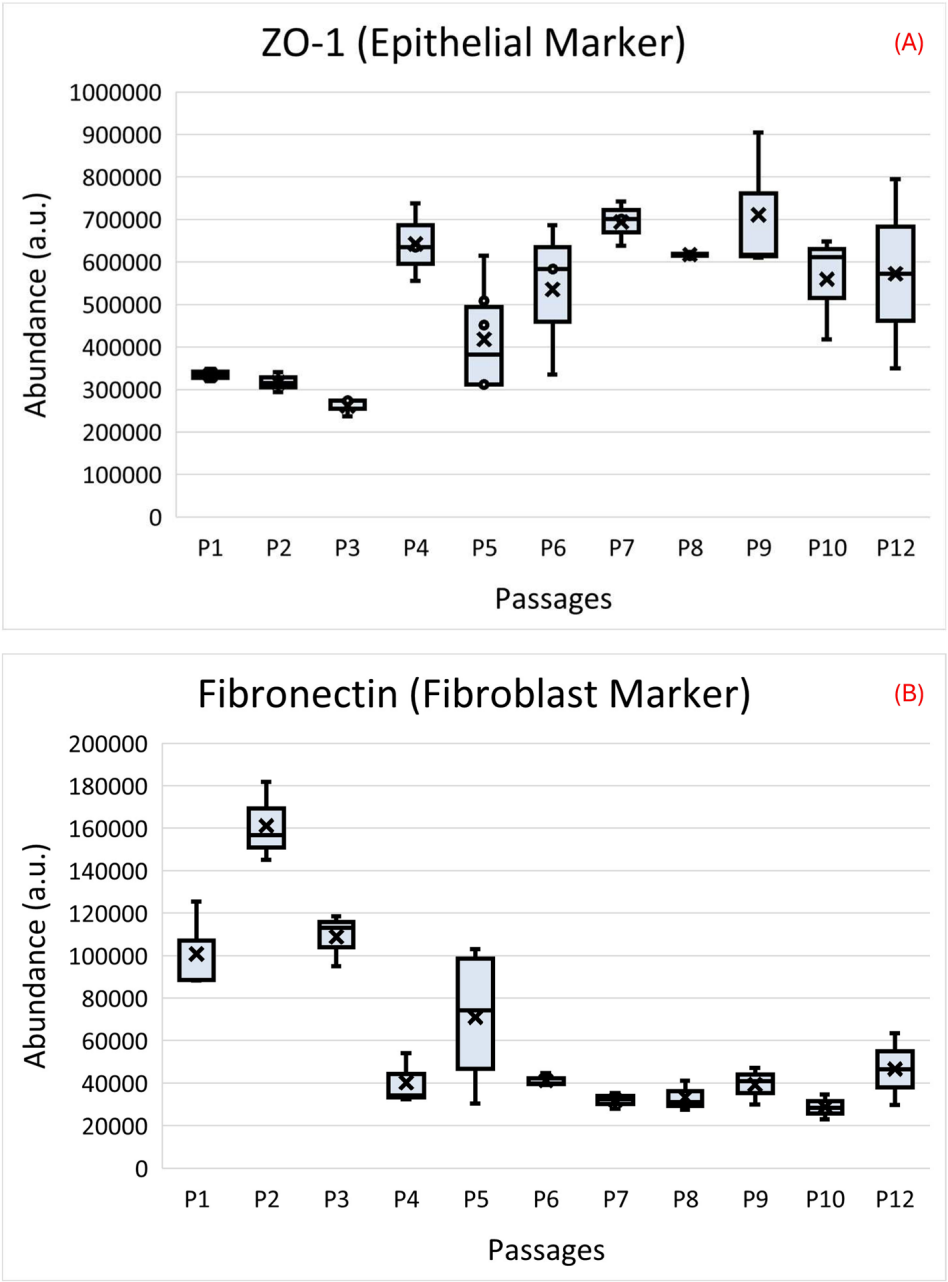
ZO-1 and Fibronectin abundance across passages in LtE-1. Boxplots of (**A**) ZO-1 (epithelial marker) and (**B**) Fibronectin (fibroblast marker) quantified from DIA-NN outputs at the protein level (DirectLFQ normalized intensities). Box = Q1-Q3 (inclusive-median quartiles), line = median, whiskers = 1.5 × IQR; outliers shown; X = mean. Points represent underlying peptide abundance per replicate. Passages are labeled relative to the primary culture

To test whether the visual shift toward epithelial-like morphology at P4 was reflected in proteomic signatures, epithelial- and fibroblast-associated proteins were examined in LtE-1 (Fig. 3; Additional file S2). Epithelial markers increased after P3: Cadherin-2 (CDH2) was 2.8-fold higher at P5 vs P1, Keratin-8 (KRT8) rose 3.2-fold, and Tight junction protein 1 (TJP1/ZO-1) was enriched at P4 (1.43-fold) and remained higher at P12 (1.71-fold). In contrast, fibroblast-associated proteins declined after P3: Fibronectin (FN1) decreased to 0.45-fold at P5, Collagen V (COL5A2) dropped to 0.40-fold at P6, and Collagen VI (COL6A1/COL6A2) remained reduced at later passages. These reciprocal trends are consistent with consolidation toward an epithelial-like state. ZO-1 is low at early passages and rises after the P3-P5 window, remaining elevated later, whereas Fibronectin is higher at early passages and declines after P4-P6.

### DIA assay library for consistent quantitation of over 3000 LtE proteins

LtE passages were quantified using a curated data-independent acquisition (DIA) assay library constructed and filtered in DIA-NN v2.2 and Skyline v25.1. The finalized LtE-1 library comprised 3,447 protein groups, 58,057 unique peptides, 60,339 precursors, and 395,011 transitions; curation retained unique proteins with ≥ 2 proteotypic peptides. The same library and identical Skyline export filters were applied uniformly to all DIA runs, including both LtE-1 and LtE-2, to ensure consistent cross-passage quantitation.

### Passage 4 marks a broad proteomic inflection point in LtE-1

**Fig. 4:**
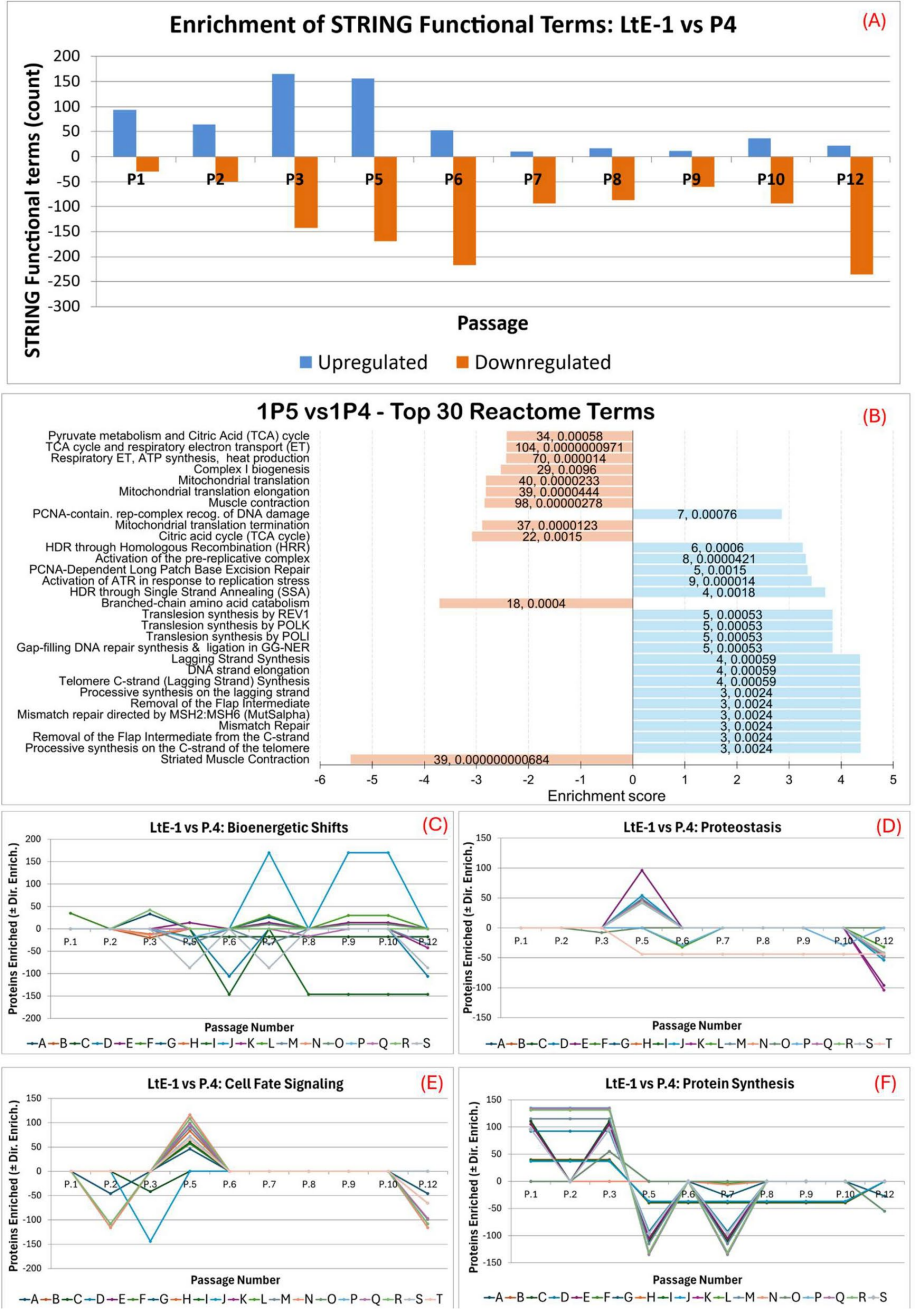
Passage-specific enrichment dynamics of LtE-1 relative to P4. **A** Overview panel summarizing enriched and depleted STRING terms for each passage vs P4 (upregulated, blue; downregulated, orange) (**B**) Reactome pathway enrichment bar charts summarizing top enriched terms (upregulated, blue; downregulated, orange) for LtE-1 P5 relative to P4. **C**-**F** Trendline graphs of representative functional groups, including cell fate signaling, protein synthesis, proteostasis, and bioenergetic shifts. Proteostasis shows a transient peak at P5, and Bioenergetics exhibits a trough at P5-P6 with a peak at P7. Definitions of letter-coded pathways used in the trendlines are provided in Additional file S4. Totals reported for each functional category reflect the combined contribution of all enriched pathways; values are category-level aggregates (signed sums of directional contributions from STRING functional terms mapped to that category and derived from the proteins assigned to those terms). The trendlines report the full passage series. As indicated by the counts bar (Fig. 4 A), the largest directional shifts cluster around the P4 window (P5-P7), and later peaks or declines are noted where present

A bar-chart overview (Fig. 4A) summarizes STRING functional terms (Gene Ontology [GO], Reactome Pathway Database [Reactome], and Kyoto Encyclopedia of Genes and Genomes [KEGG]) by passage relative to P4 to summarize the main functional changes across the series. Here, terms refer to individual functional term entries (e.g., KEGG: Ribosome; Reactome: translation initiation/elongation/rRNA processing), and categories are umbrella groups (e.g., Protein synthesis, RNA processing, Adhesion); category aggregate denotes the summed contribution of terms within a category for a given comparison. Early passages showed moderate enrichment with fewer depletions. P1 (94 upregulated/30 downregulated) emphasized differences in matrix/adhesion and secretory targeting, and P2 (64/50) tilted toward differences in translation/mitochondrial translation and nonsense-mediated decay (NMD) relative to P4. P3 (165/143) was dominated by translation/NMD and ribosome biogenesis. These molecular signatures are consistent with elevated translation-associated enrichment during early passages (and accompanying protein synthesis) as well as cell adhesion during early passages preceding the transition window. Full lists for all enriched functions and pathways are available in Additional file S3.

Immediately following the P4 inflection point, P5 (156/169) displayed increases in DNA replication and repair modules, including telomere C-strand and lagging-strand synthesis with mismatch repair, accompanied by declines in oxidative phosphorylation and tricarboxylic acid (TCA)-cycle relative to P4. This signature is consistent with increased representation of DNA damage response and integrity pathways and reduced representation of oxidative metabolism during the transient growth slowdown inferred from growth kinetics. By P6 (53/217), rRNA regulation and DNA-repair terms had receded, with lipid/steroid biosynthesis and lysosome among the few enriched sets. Taken together, the P4–P6 transition window includes a transient DNA replication/repair signature at P5 that diminishes by P6, alongside changes consistent with membrane lipid and protein turnover.

Later passages exhibited comparatively few upregulated terms and many downregulated functions across P7 (10/93), P8 (17/87), P9 (12/60), and P10 (36/94). P7-P10 also exhibited upregulation of intermittent cholesterol/steroid biosynthesis alongside declines in contractile programs and mitochondrial amino-acid catabolism. This trend is consistent with continued remodeling of membrane lipid and protein metabolism after the transition window. By P12 (22/235), enrichment shifted towards apoptosis-associated DNA fragmentation and peroxisome proliferator-activated receptor alpha (PPARα)-linked lipid regulation, with depletion across TCA/electron transport chain (ETC) and ATP-coupling modules. This signature suggests continued remodeling by P12, rather than a fully stabilized endpoint, consistent with the possibility of ongoing selection beyond the passages sampled here. Cytoskeletal/adhesion remodeling was emphasized during early passages and then recedes as DNA replication, DNA/RNA repair and lipid, protein, and mitochondrial energy metabolism reorganize around the P4 inflection point. The largest changes occur between P5 and P7 (Fig. 4A). To illustrate these changes, a representative term-composition visualization is provided for P5 (Fig. 4B). Functional category dynamics across all passages indicate increased changes in bioenergetic functions after passage 4, peaks in remodeling proteostasis and cell fate signaling at passages 5 and 12, and an inflection from enrichment to depletion of protein synthesis functions at P4 followed by stabilization after P8 (Fig. 4C-F). Full term lists and scores for each comparison are provided in Additional file S4.

Protein synthesis peaked at P3 (category aggregate of + 1,386; being the sum of mapped proteins upregulated across all translation terms) then sharply decreased at P5 (category aggregate; 1,276 downregulated) (Fig. 4F). Ribosome biogenesis (82 mapped proteins) and translation initiation (74 mapped proteins) dominated the pre-P4 peak, while rRNA processing (135 downregulated mapped proteins at P5) contributed strongly to the post-P4 decline. After the reversal, the protein-synthesis category aggregate remained negative at later passages, including P7 (− 1,319) and P12 (− 137) (Fig. 4F). Proteostasis peaked immediately after transition (P5 category aggregate: 1,035 upregulated across folding and degradation modules), reflecting enrichment in antigen processing/ubiquitination and proteasome degradation (96 upregulated mapped proteins) and Proteasome (44 upregulated mapped proteins). Pathway-level contributions included degradation of β-catenin by the destruction complex (54 upregulated mapped proteins) and anaphase-promoting complex/cyclosome (APC/C)-mediated degradation of cell-cycle proteins (47 upregulated mapped proteins) at P5 (Fig. 4D). By P7, the proteostasis category aggregate had shifted negative (net − 882), and by late passages it declined further (P12: 1,320 downregulated), with losses spread across folding-related terms such as chaperonins (45 mapped proteins) and ER-associated degradation (ERAD) components (34 mapped proteins) (Fig. 4D). These pervasive changes in proteostasis and protein synthesis indicate significant shifts in these processes from before to after P4 metabolism. This observation indicates a change in how sustained cell proliferation after the transition window is supported by protein synthesis compared to the initial burst proliferation in early passages preceding the transition window.

Sustained cell proliferation after resumption of faster growth following the transition window is supported by remodeling of bioenergetics functions that peaked at P7 (250 upregulated, 183 downregulated; net + 67 mapped proteins), with oxidative phosphorylation (87 mapped proteins) and respiratory electron transport as principal upregulated contributors (Fig. 4C). An earlier uptick was present at P3 (net + 48), troughs occurred across P5-P6, modest positives appeared at P9-P10 (net + 22, + 16), and a marked decline was evident by P12 (− 429) (Fig. 4C). Metabolic pathways were prominently regulated across passages, with a peak mirroring the bioenergetics peak at P7 and tapering by P12 (Fig. 4C). The metabolic pathway signature was dominated by lipid metabolism (170 mapped proteins) and amino acid metabolism (146 mapped proteins) alongside carbon metabolism (106 proteins) and pyruvate/TCA cycle modules (34 mapped proteins). These metabolic pathway patterns reflect sustained remodeling of energetic and biosynthetic programs beyond the transient translational peak.

Cell fate signaling modules also showed transient increases after the P4 inflection point. At P5, mitogen-activated protein kinase (MAPK) family signaling cascades (116 upregulated mapped proteins), protein kinase B (AKT) signaling (83 upregulated mapped proteins), and Wnt/WNT-β-catenin (WNT) dependent transcriptional regulation (72 upregulated mapped proteins) exhibited strong enrichment, often coinciding with noncanonical nuclear factor kappa-B (NF-κB) and RAF/MAPK cascades. Across the series, the cell-fate signaling category aggregates show a major positive net increase at P5 (+ 1,722), a strong elevation at P6 (+ 993), a smaller reinforcement at P7 (+ 139), a return toward baseline at P8-P10, and a late decline at P12 (− 1,137) (Fig. 4E; Additional file S4). These signaling signatures support changes in cell survival signaling and cell adhesion, given that MAPK, WNT, AKT, and NF-κB signaling pathways are major regulators of these functions. Cell-cycle remodeling was also most prominent around the P4 inflection point, further supporting changes in cell survival and turnover. At P5, mitotic metaphase and anaphase modules were enriched with 121 mapped proteins. Remodeling of cell-cycle signals weakened thereafter, indicating stabilization (Additional file S4).

DNA repair pathways were modestly but distinctly upregulated during the P4 inflection point. At P5, mismatch repair was supported by five mapped proteins, and gap-filling repair modules showed similar contributions (three to five mapped proteins each). Although numerically smaller than translational or metabolic categories, these pathways highlight transiently increased genome-maintenance activity and support the overall trend of altered cell survival signaling near the P4 inflection point (Additional file S4). For context relative to the original primary culture, the complete LtE-1 longitudinal series versus P1 is provided in Additional file S5 and Additional file S4.

### Parallel proteome changes across the P4-P7 spanning the transition window and early post-transition passages in LtE-2 but with distinct trajectories compared to LtE-1

**Fig. 5:**
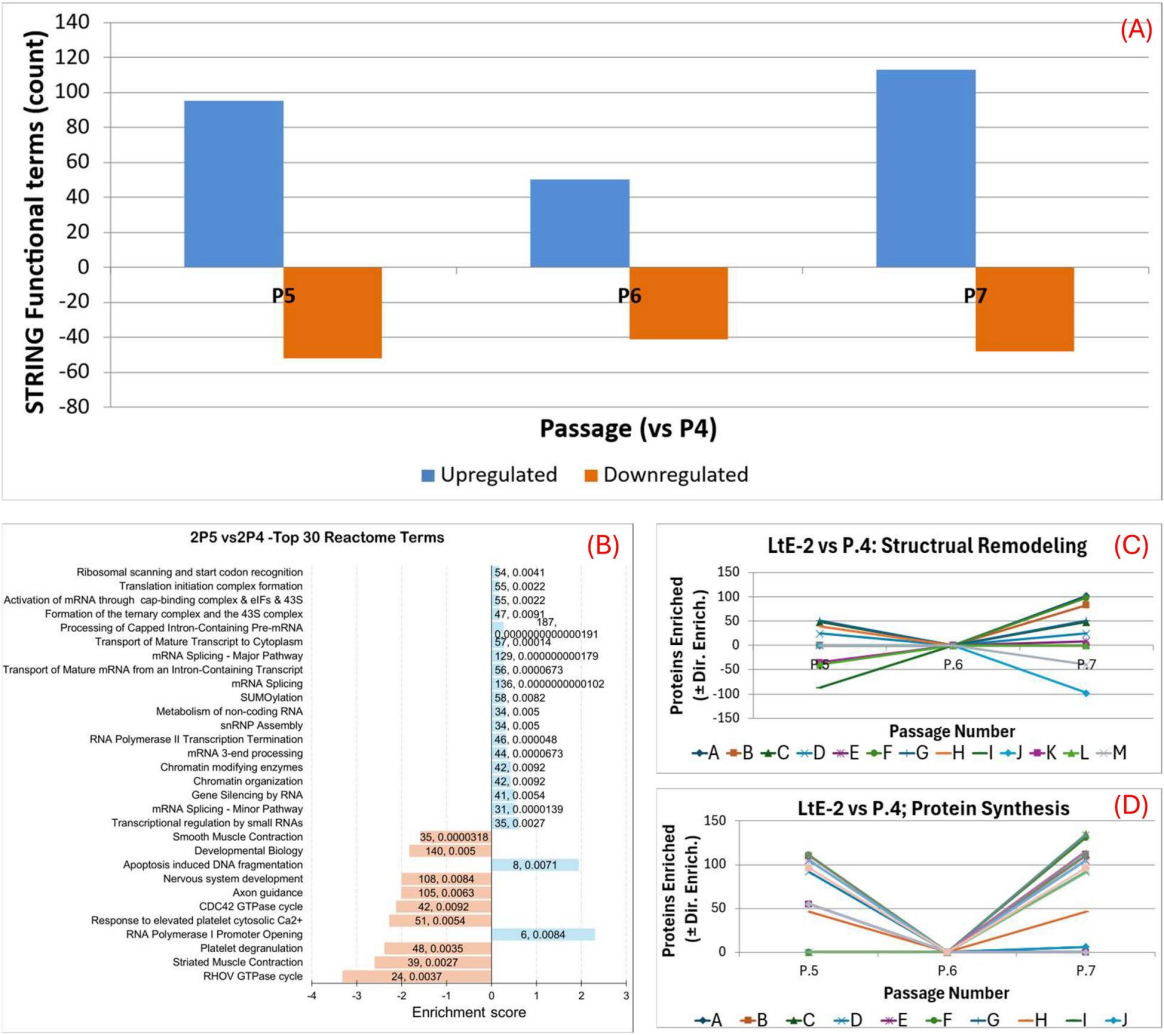
Passage-specific enrichment dynamics of LtE-2 relative to P4. **A** Overview panel summarizing STRING terms for each passage vs P4 (upregulated, blue; downregulated, orange). **B** Reactome bar chart showing the distribution of top enriched pathways for LtE-2 passages compared with P4. **C**-**D** The trendlines in Fig. 5C-D show category-level aggregates, i.e., signed sums of mapped protein contributions across STRING terms within each category. Trendline graphs of major functional categories highlight enrichment of protein synthesis and structural remodeling. Trendlines are centered at P4; values at P5-P7 reflect net positive enrichment relative to P4. Dips indicate term-level fluctuations and do not imply loss of the aggregate signal. Both categories show elevation across passages. Definitions of letter-coded pathways used in trendline graphs are provided in Additional file S8

LtE-2 exhibited similar but also distinct aspects of proteome dynamics as LtE-1, with the most striking changes also occurring around passage 4 (Supplementary Table S3). The number of STRING functional terms (i.e., individual GO/Reactome/KEGG entries) enriched or depleted (vs P4) are displayed in Fig. 5A. Relative to P4, LtE-2 shows strong proteome remodeling at P5 (95 enriched, 52 depleted), decreased remodeling at P6 (50 enriched, 41 depleted) and renewed reinforcement at P7 (113 enriched, 48 depleted) (Fig. 5A). A representative term-composition view for P5 is shown in Fig. 5B. Functionally, this remodeling is dominated by RNA-processing/splicing and focal-adhesion/extracellular matrix (ECM) remodeling, with adhesion signals persisting across P5-P7 (Fig. 5C-D). Across the window, the total numbers were 149 terms at P5 and 161 at P7.

Trendline analyses confirmed sustained activation of translation and RNA processing relative to P4 (Fig. 5C-D; Table S3 and S6). Here, “category aggregate” denotes the signed sum of mapped protein contributions across STRING terms within a category for a given passage comparison (vs P4); up/down term counts per passage are shown in Fig. 5A. Protein synthesis was upregulated at P7, with a category aggregate of 1623 proteins across translation-related terms (e.g., KEGG Ribosome; Reactome translation initiation/elongation/rRNA processing). Ribosome (KEGG; 115 mapped proteins) was a major upregulated contributor. RNA processing also increased with passage number (P5: category aggregate 828; P7: 766 category aggregate), dominated by mRNA splicing entries (category aggregate 136). Adhesion-related terms remained upregulated after P4; focal adhesion (KEGG; 102 mapped proteins) contributed strongly to the upregulated adhesion category aggregate at P7. Although several individual terms fluctuated between passages, the category-level signals for protein synthesis, RNA processing, and adhesion remained upregulated at each stage (Additional file S6).

Transcriptional regulation was modestly but consistently enriched following P4. At P5, chromatin-modifying enzymes (42 upregulated mapped proteins) and mRNA splicing (category aggregate + 136) dominated, while at P7 RNA Polymerase I promoter activities increased (6–20 enriched terms associated with promoter clearance, opening, and transcription). These patterns highlight targeted reinforcement of chromatin remodeling, transcriptional regulation, and RNA processing complementing the more persistent protein-synthesis and cell-adhesion remodeling programs.

Stress-responsive signaling also underwent significant changes in LtE-2, including apoptosis-related functions that were upregulated at P5 (8 mapped proteins). Although less pronounced than metabolic or translational enrichments, these terms indicate remodeling of transient cell integrity surveillance mechanisms during the transition (Table S3). Complete lists of significant STRING terms for LtE-2 passages, including direction (enriched/depleted) for each comparison vs P4, are provided in Supplementary Table S3.

In summary, LtE-2 showed strong persistent reinforcement of RNA processing and cell-adhesion programs. Thus, in LtE-2, traversing the transition window and the corresponding growth slowdown is defined by continuous strengthening of RNA-processing and cell adhesion programs, with stress-surveillance modules contributing to a balanced proliferative state. These processes were also associated with the P4 inflection point in LtE-1 during early establishment. However, in contrast to LtE-1, remodeling of translation and proteostasis was less pronounced in LtE-2, underscoring that routes to continuous cell line establishment were distinct but converged in shared cell functions (cell adhesion, integrity surveillance, and translational preference control).

### LtE-1 protein interaction subnetworks directly before and after the P4 transition

**Fig. 6:**
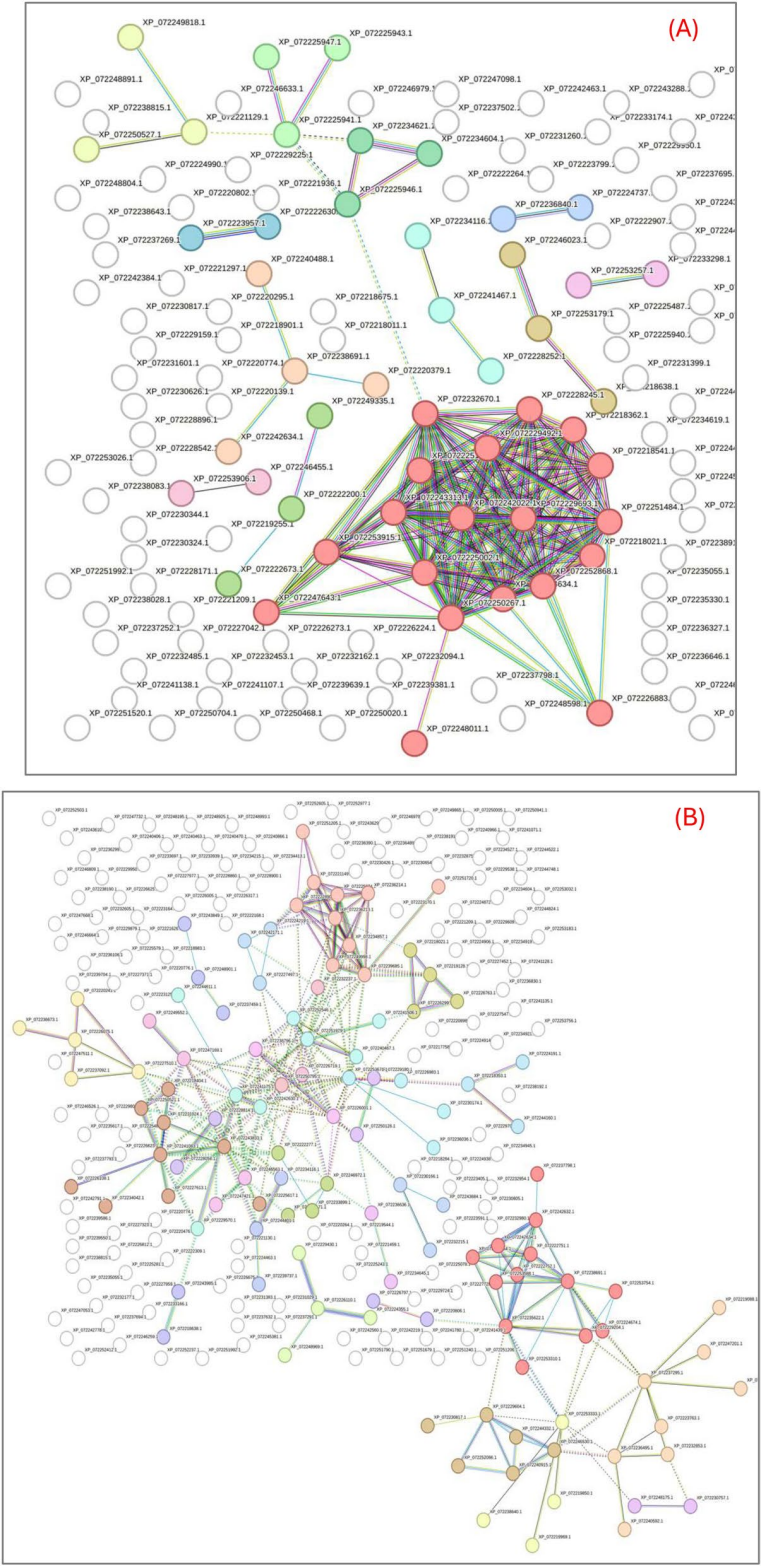
STRING MCL subnetworks before and after the P4 transition in LtE-1. **A** Pre-P4: STRING network generated from proteins recurrently altered in P1-P3 vs P4 (up defined as FC > 2, down as FC < 0.5; recurrence ≥ 2 passages). **B** Post-P4: STRING network from proteins recurrently altered in P5-P7 vs P4 (same thresholds). Networks were constructed in STRING for *Leuresthes tenuis* at interaction score ≥ 0.400 (medium) with MCL clustering (inflation = 3). Nodes are grouped and colored by MCL clusters; edges represent STRING interactions at the specified confidence, see Table 1 for annotations. Cluster identities/annotations are taken directly from STRING; interactive STRING views for the pre-P4 and post-P4 subnetworks are available as Refs. 90 and 91, respectively

To evaluate whether interaction modules exhibit coherent regulation across the P4 transition pivot in LtE-1, STRING subnetworks were built from the accession numbers of recurrence-filtered proteins (recurrence ≥ 2 passages; up defined as FC > 2, down as FC < 0.5) [27]. Pre-P4 (P1-P3 vs P4) and post-P4 (P5-P7 vs P4) lists were analyzed separately using the STRING multi-protein input option and MCL cluster analysis (organism = *L. tenuis*, interaction score ≥ 0.400; MCL inflation = 3) (Fig. 6; Table 1).

**Table 1:**
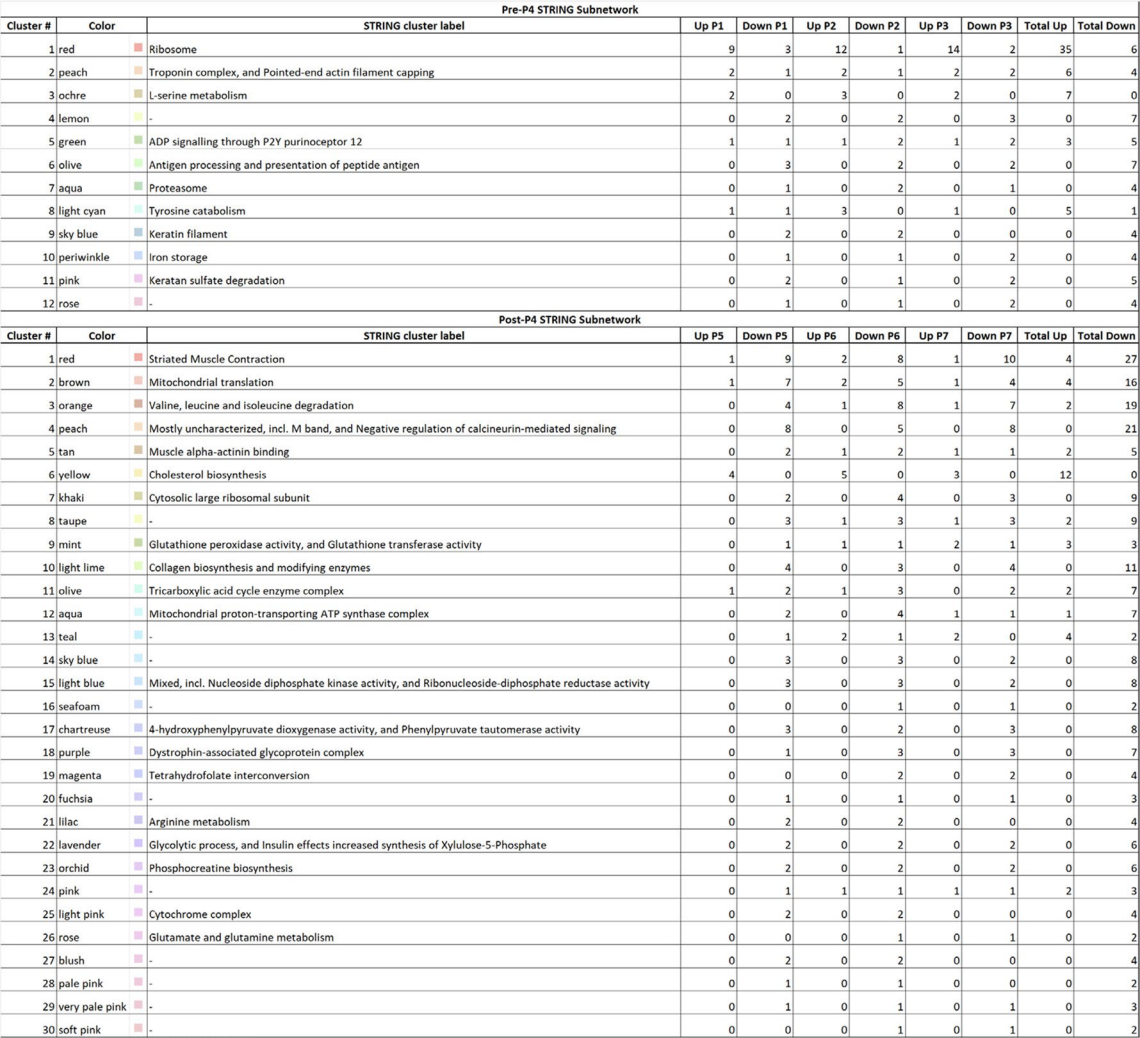
Numbers of significantly up-/and down-regulated proteins function groups by STRING MCL cluster before and after the P4 transition for LtE-1

Proteins recurrently altered relative to P4 (recurrence ≥ 2 passages; up = FC > 2; down = FC < 0.5) were analyzed in STRING for *Leuresthes tenuis* (interaction score ≥ 0.400; MCL inflation = 3). For each MCL Cluster #, the STRING cluster label and number of up/down proteins per passage are reported for Pre-P4 (P1-P3 vs P4) and Post-P4 (P5-P7 vs P4), with phase totals at right. Clusters without a STRING primary description (“-”) and any unassigned proteins are provided in Additional file S7. This table corresponds to the subnetworks shown in Fig. 6

Eight clusters associated with proteostasis functions are represented mostly by upregulated, and some down-regulated, proteins during passages preceding P4, indicating that remodeling of these functions takes priority prior to the transition window (Table 1). The largest functional cluster is characterized by proteins representing ribosomal pathways (Cluster 1; red nodes in Fig. 6A). This Cluster 1 module provides a unifying axis for the pre-P4 state, standing in contrast to the post-P4 network where it is not consolidated (Fig. 6A). Proteasome (Cluster 7; aqua nodes) and antigen processing/presentation of peptide antigen (Cluster 6; olive nodes) are also present in the pre-P4 network, consistent with early proteostasis engagement. The pre-P4 network contains fewer multi-node clusters and fewer upregulated proteins overall than the post-P4 network (Table 1; Fig. 6). Where ECM/adhesion and contractile clusters appear, such as the keratin filament module (Cluster 9; light-blue nodes), they already show downregulation in one or more pre-P4 passages, whereas a coherent cholesterol/sterol lipid metabolism module is not evident before P4 (Fig. 6A; Table 1).

The post-P4 network includes multiple clusters indicative of lipid metabolism and cholesterol/sterol biosynthesis that were predominantly upregulated, such as cholesterol biosynthesis (Cluster 6; yellow nodes) and mitochondrial proton-transporting ATP synthase complex (Cluster 12; aqua nodes). These modules are consistently associated with mostly upregulated proteins across P5-P7 (Fig. 6B; Table 1). Additionally, multiple ECM/collagen processing and contractile/striated-muscle clusters are predominantly represented by down-regulated proteins across post-P4 passages, including collagen biosynthesis and modifying enzymes (Cluster 10; light-lime nodes) and the abundant striated-muscle contraction module (Cluster 1; red nodes). Additional metabolic modules, such as branched-chain amino-acid degradation (Cluster 3; orange nodes), are also represented primarily by downregulated proteins after P4 (Fig. 6B; Table 1). These network-level patterns reinforce the notion that post-P4 passages in LtE-1 are dominated by remodeling of membrane lipid and protein metabolism together with reorganization of cytoskeletal/contractile and adhesion/ECM-associated modules (Fig. 6; Table 1).

## Discussion

*Leuresthes tenuis* is distinctive among teleost sources used for cell culture because embryos develop terrestrially in beach sand for an extended interval until hatching is environmentally triggered (e.g., by agitation in seawater). Grunion embryos develop in the sand until they are competent to hatch, and then maintain a relatively stable metabolic rate but little to no further development until triggered to hatch [22, 28]. This unusual early-life history, together with repeated observations that embryo-derived grunion cultures rapidly progress to sustained proliferation with only brief interruptions during early establishment, motivated a passage-resolved analysis of establishment dynamics in vitro. Under the present conditions, establishment of continuously replicating cell lines in *L. tenuis* repeatedly presented as staged rather than gradual, with passage (P) 4 delineating the shift from transient surges to sustained regulatory control of cell proliferation. By comparing two independent California grunion embryonic-derived cell lines established by distinct individual fish, LtE-1 and LtE-2, shared proteomic biosignatures and stochastic features that converge on continuous-proliferation phenotypes were identified. Because practical constraints restrict early-passage sampling while continuous cell cultures are being sustained, high-sensitivity DIA proteomics (DIA-NN, Skyline, DirectLFQ) was utilized to enable confident quantitation from small inputs, which made it possible to resolve passage-specific signals around the P4 transition point that would otherwise be missed. This comparative analysis illustrates that cell establishment in vitro can exhibit reproducible, passage-linked proteome dynamics that can be traced through enrichment profiles and interaction-module structure [29]. Because early cultures contained mixed morphologies and cell population proteomics integrates across subpopulations, passage-linked patterns are interpreted here as establishment-associated culture dynamics rather than lineage-resolved evidence of cell-intrinsic mechanism. For both LtE-1 and LtE-2 cell lines, P1-P3 denote early establishment/mixed morphologies; P4 is the operational pivot; P4-P6 capture the transient slowdown; P7 + represent post-transition sustained growth under our culture conditions.

Independent growth measurements support the P4 pivot (inflection point) and define a P4-P6 transition window marked by a transient slowing of proliferation (senescence-like) followed by resumed growth. Senescence was not independently assayed (e.g., by SA-β-Gal staining), given reported variability in SA-β-Gal performance across fish cell lines [30, 31]. Therefore, the term senescence-like is used here to describe the transient growth slowdown inferred from time to ~ 80% confluency (t80) and population doubling (PD) trajectories. Both cell lines exhibited a brief growth slow-down that aligned with the P4-P6 transition window, after which faster proliferation resumed. Morphologically, the abundance of fibroblast-like cells that were present at early passages diminished around this window, while epithelial-like cells became dominant in the culture. Accordingly, apparent pathway-level shifts across early passages can reflect changing population composition as well as cell-intrinsic remodeling within surviving subpopulations. Proteomic markers were directionally consistent with this interpretation: epithelial-associated proteins such as TJP1/ZO-1 and KRT8 rose in abundance after P3, while fibroblast-associated proteins (e.g., Fibronectin, COL5A2/COL6A1) declined [32, 33]. These reciprocal marker trends align with the visual observation of epithelial consolidation at P4 (Fig. 1). One candidate explanation for this shift is a mesenchymal-to-epithelial transition (MET), a cell-state change that has been described in multiple systems during acquisition of stable epithelial-like growth in vitro [34–37]. However, the present cell population proteomics design cannot distinguish MET from selective expansion of pre-existing epithelial-like subpopulations and/or selective loss of fibroblast-like subpopulations [36, 38]. The brief t80 (time to ~ 80% confluency) elevation near the pivot, together with the transient reinforcement of DNA-repair and telomere-maintenance near P4, indicates checkpoint surveillance and possible bypass during the transition into continuous growth, aligning with gatekeeper models and followed by attenuation at later passages [11, 39–41]. Because these DNA-repair and telomere signals peak near P4 and attenuate thereafter, they may serve as candidate biomarker targets for bypassing checkpoints to achieve immortalization. Antibody-based methods for abundance measurements of single predetermined protein markers were not used in this study because it is known that direct modern mass spectrometry approaches are more accurate, have higher selectivity, and are overall superior for protein quantification compared with antibody-based methods [42–45]. Moreover, assaying such markers at the transcript level is less reliable than at the protein level because of known non-linearity between these levels and closer proximity of the proteome to ultimate phenotype [46–50].

In LtE-1, the P4 transition featured sharp and temporary shifts in functional enrichment of protein synthesis followed by depletion at P5 and stabilization over subsequent passages. This trend corresponds to a temporary decrease in cell proliferation (increase in t80) up to P6 before resuming in later passages after recovering from a transient growth slowdown (senescence-like interval). Stalled cell proliferation is a hallmark of senescence in many species and cell types [51–55], which, in these *L. tenuis* cultures, seems to coincide with the transient slowdown and altered regulation of protein synthesis. In parallel, proteostasis regulation peaked at P5, was downregulated by P7, and remained reduced at P12, indicating that corresponding quality control (QC) checkpoints are de-emphasized past P6. Temporally, the proteostasis peak brackets the local t80 maximum within the transition window and resolves as t80 shortens, indicating short interval buffering rather than a durable program (Fig. 2). This pattern indicates brief engagement of stress-buffering during the immediate transition, in line with unfolded-protein-response and broader proteostasis quality control programs that stabilize cells over short intervals before receding as growth control consolidates [56–61]. This proteome reversal from pre- to post-P4 patterns may indicate activation of stress-buffering or -response mechanisms during the transition window of brief senescence-like growth slowdown to support cell line establishment. This conclusion was supported by corresponding enriched functions observed in both LtE-1 and LtE-2 cell lines and prior studies [53, 56–61]. Reinforcement and rebalancing of bioenergetic and lipid/protein metabolic functions during post-P4 passages were prominent features in both LtE-1 and LtE-2 lines and are consistent with metabolic remodeling reported during prolonged proliferation and culture adaptation in other systems, including non-transformed cells adapting to sustained proliferation in vitro [62, 63]. While many mechanistic frameworks for metabolic remodeling are best developed in tumor contexts, similar remodeling has also been reported during prolonged proliferation and culture adaptation in non-transformed cells [64, 65]. Early adaptation to media composition and nutrient availability remains a plausible, non-exclusive contributor to the observed lipid, bioenergetic, and proteostatic patterns [66]. Immediately after P4 in LtE-1, transient elevations in MAPK, AKT, WNT, and NF-κB cascades also indicate checkpoint traversal at the P4 transition point through the engagement of canonical drivers of cell-cycle entry, survival, and proliferative competence [67–72]. Placed alongside the short-lived proteostasis regulation peak and subsequent decline, these regulatory kinase increases further support a surge-based passage through the transition window that resolves as growth control stabilizes.Collectively, these complementary proteomics signatures provide molecular insight into how LtE-1 establishment-associated remodeling (a growth slowdown centered on the P4 inflection point) is marked by transient remodeling of translation/protein synthesis, proteostasis, and cell-fate signaling, followed by stabilization of membrane lipid and protein metabolic programs and continued remodeling of adhesion/ECM-associated and cytoskeletal features. Together these results support the cell proliferation data suggesting that passage 4 marks a staged transition in LtE-1, where short-lived surges in these growth-linked categories give way to durable metabolic and structural remodeling that are indicative of passing the transition window (Fig. 4A, C-F). Because proteomics was quantified through P12, continued selection and additional proteome change beyond P12 cannot be excluded despite shortening of proliferation trajectories after the transition window.

MCL clustered interaction networks built from recurrence-filtered proteins contextualize the P4 transition point in LtE-1. In the pre-P4 subnetwork (P1-P3 vs P4), the ribosome-associated cluster is prominent, consistent with early translational engagement at the brink of transitioning from pre- to post-transition point proliferation. The topology shows fewer multi-node clusters, with ECM/adhesion or contractile clusters already containing downregulated nodes in one or more passages. Post-P4 (P5-P7 vs P4) in LtE-1, the architecture consolidates and dominates around a sterol/cholesterol biosynthesis module with predominantly upregulated nodes, while ECM/collagen and contractile modules, together with additional metabolic clusters (for example, branched-chain amino-acid degradation), trend towards downregulation. This pre/post transition point pattern indicates a shift toward membrane and energy biosynthesis as growth stabilizes, and a concurrent reduction in cytoskeletal restraint, in line with studies associating lipid/sterol synthesis with proliferative demand and attenuated contractile tone with sustained growth [32, 33, 62, 63]. In LtE-2, RNA processing and translation categories (promoting protein synthesis) remained upregulated across P5-P7, indicating that this cell line sustains biosynthetic programs through the transition window and into early post-transition passages with passage-to-passage variation to support proliferative capacity and genome integrity [56, 57]. Independent growth metrics mirrored this pattern, with a brief t80 peak at P5-P6 followed by shortening of t80 by P7-P8. This sustained enrichment aligns with evidence that altered splicing supports proliferative capacity and genome stability, indicating that RNA-processing factors help maintain continuous growth within the transition window and may serve as biomarkers and potential regulators of continuous growth [57, 58]. Adhesion/ECM terms show mixed responses at the individual-term level, but the category aggregate remained upregulated and persisted at P7. For example, focal adhesion is reinforced at P7 relative to P4 in LtE-2, illustrating later-passage strengthening within the adhesion/ECM module while the aggregate persists despite term-level oscillation. Together, adhesion/ECM pathway regulation indicated ongoing structural remodeling and cell-substrate signaling consistent with integrin-ECM mechanotransduction and an important role of Yes-associated protein (YAP) and transcriptional co-activator with PDZ-binding motif (TAZ) in Hippo pathway regulation of cell proliferation [33, 60, 61, 73, 74]. The modest enrichment detected around the transition window for apoptosis-linked terms indicates transient stress surveillance, quality control, and checkpoint engagement before proliferative control stabilizes after the P4 transition point, consistent with stress-response buffering observed during senescence-like transitions or growth slowdowns [52–55]. Given the established roles of YAP/TAZ as Hippo-pathway effectors in proliferation, persistent adhesion/ECM signaling identifies reinforcement of cell–matrix communication as a potential intervention point. Together, these features indicate that LtE-2 traverses the P4-P6 transition window by maintaining and reinforcing biosynthetic and adhesion programs to support continuous growth into P7 [57, 60–62, 73, 74]. Across both LtE-1 and LtE-2, the transition window is characterized by coordinated remodeling in categories linked to biosynthesis (RNA processing/translation), proteostasis quality control, and adhesion/ECM-associated structural signaling, coincident with consolidation toward epithelial-like morphology. However, the temporal trajectories differ: LtE-1 is dominated by sharp, transient surges and reversals across adjacent passages, whereas LtE-2 shows more sustained reinforcement of RNA processing/translation with oscillatory term-level behavior. These shared categories, coupled with divergent dynamics, support a model in which establishment involves convergence on similar functional endpoints through line-specific trajectories.

The morphology and t80 trajectories after P4 in both lines, together with consolidation of biosynthetic, proteostatic, and adhesion programs, are consistent with selection and competition dynamics during establishment, in which subpopulations that resume proliferation after the transition window expand. Such dynamics are well documented during immortalization [11, 13, 75]. However, cell population based assays cannot resolve whether these dynamics reflect selection, cell-intrinsic state transitions, or a combination of processes. Discriminating among selection, cell-intrinsic state transitions, and media adaptation will require lineage-resolved profiling, including single-cell and multi-omic approaches across P3–P7 [76].

Considered jointly, LtE-1 and LtE-2 establishment trajectories, although stochastic and different in many details, converge on stabilized cell proliferation and epithelial-like morphology after the P4 transition point. The transient proteostasis regulation peak associated with the transition window, most prominently in LtE-1, supports a testable hypothesis that short-interval manipulation of proteostasis quality control and checkpoint regulation facilitates passage through the transition period in cultures that would otherwise stall [56–61]. In parallel, the persistence of adhesion/ECM signaling, particularly in LtE-2, indicates that reinforcement of cell–matrix communication may be leveraged to maintain proliferative competence, consistent with a role of integrin-mediated mechanotransduction via YAP/TAZ in sustaining cell proliferation [33, 60, 61, 73, 74]. Accordingly, manipulating the extracellular environment (substrate stiffness, matrix engagement, integrin signaling) or modulating these pathways could be explored to facilitate immortalization of primary cells in culture [73, 77–79].

Fish cell line establishment has most often been documented descriptively, and many teleost lines are reported as fibroblast-like or mixed early in derivation. In this context, California grunion cultures are notable for reproducible convergence on epithelial-like morphology across independent derivations, alongside coordinated remodeling in adhesion/ECM, cytoskeletal, and related structural programs. These features motivate future cross-species comparisons to determine which aspects of the transition window reflect shared constraints of culture establishment versus lineage- or tissue-specific trajectories. The epithelial-like endpoint reported here is consistent with several widely used teleost lines that are routinely described as epithelial, including the Chinook salmon embryo-derived CHSE-214 line [80]. CHSE-214 and other embryo/larval-derived teleost cultures are often maintained as epithelial-like monolayers after establishment, supporting epithelial-like monolayers as a frequent endpoint of culture selection and stabilization rather than a lineage-specific anomaly [15, 81].

## Conclusions

The early passage proteomic landscapes of two independently derived *Leuresthes tenuis* embryonic-derived cell lines underscore both divergence and convergence in routes to continuous cell line establishment. Across LtE-1 and LtE-2, P4 transition point associated remodeling emerged as the consistent signature. Checkpoint-associated signaling and proteostasis quality control regulation were prominent proteomic signatures associated with the P4-transition-point. Despite considerable differences and stochastic features associated with proteome changes during early cell line establishment, both LtE lines converged on sustained proliferation with epithelial-like morphology by the post-transition passages sampled here. The morphological convergence is corroborated by consistent proteomic markers, with increases in epithelial-associated proteins and decreases in fibroblast-associated proteins after the transition window. Recurrence of these features in LtE-1 and LtE-2, and similar morphological changes in yet another independently derived cell line (LtE-3), support the reproducibility of this establishment-associated pattern in grunion embryonic-derived cells under the present conditions. Because early cultures contained mixed morphologies and these are proteome measurements of cell populations, the present data define passage-resolved establishment-associated biosignatures but cannot resolve lineage (e.g., MET versus selective outgrowth) without lineage-resolved approaches. Cell proliferation measurements indicate a consistently brief transition window that is readily overcome by grunion embryonic-derived primary cultures in the same window, making them useful models for studies of California grunion cell line establishment and early passage dynamics. Taken together, the observed proteomic changes in translation, RNA processing, cell adhesion, bioenergetics, and proteostasis define a set of biosignatures associated with the transition window and subsequent sustained growth. These biosignatures can serve as candidate readouts for recognizing the onset of continuous growth and prioritize testable targets for future cross-species validation. They provide indicators for anticipating the approach to continuous proliferation and suggest actionable points in proteostasis, adhesion signaling, and transition-associated checkpoint signaling. More broadly, the contrast between pre-P4 and post-P4 subnetworks captures the shift to a post-transition architecture with consolidated sterol biosynthesis and attenuated ECM/collagen processing and contractile modules, a configuration that parallels the proteomic and morphological consolidation of continuous growth.

## Methods

### Collection

California grunion (*Leuresthes tenuis*) eggs were collected by hand and artificially fertilized at Doheny Beach (LtE-1, LtE-2) and Cabrillo Beach (LtE-3), California. Adults were hand-captured, kept in 5-gallon buckets filled with natural seawater, and gently massaged to release gametes into plastic cups [82, 83]. Milt from three males was added to eggs from one female, and gametes were mixed with 32 parts-per-thousand (ppt) artificial seawater (Red Sea Salt, Red Sea, Houston, TX, USA). After a 1 h incubation, embryos were rinsed three times with artificial seawater and transferred to incubation chambers composed of Ziploc containers filled with sterilized beach sand, cloth material, and paper towels [28]. Eggs were maintained at 25–27 °C in darkness for 6 weeks in the UC Davis Cole B Animal Facility until hatching competent (i.e., fully developed embryos capable of hatching under standard induction conditions).

### Derivation of California grunion cells (LtE-1, LtE-2, and LtE-3)

At 6 weeks post-fertilization, embryos were hatched by mechanical agitation in seawater [22]. A single yolk-sac hatchlingwas randomly selected for each cell line. Larvae were surface-sterilized by sequential 70% ethanol (30 s) and 4% bleach (30 s) dips, blotted on KimWipes, and minced into ~ 1 mm fragments with sterile razor blades [84]. Tissue fragments were rinsed sequentially in three dishes of sterile phosphate-buffered saline (PBS). All fragments from the single minced hatchling were plated into a single 35 mm dish per linecontaining a thin film of complete medium (Leibovitz’s L-15 supplemented with penicillin 100 U/mL, streptomycin 100 µg/mL, gentamicin 50 µg/mL, epidermal growth factor 10 ng/mL, and 20% fetal bovine serum (FBS) for LtE-1 and 10% FBS for LtE-2 and LtE-3). The thin film was prepared by adding ~ 1 mL medium and then withdrawing most of it with the dish slightly tilted, leaving enough (~ 100–200 µL) to keep tissue explants moist while promoting adherence. Explants were incubated at 28 °C overnight at ambient CO₂. On the following day, fresh medium (3 mL) was added dropwise to minimize disturbance with amphotericin B (12.5 µg/mL) added to explant dishes to prevent contamination. Incubation conditions were maintained at 28 °C at ambient CO₂ for the duration of the experiment. Before reaching confluency, weekly partial medium exchanges (~ 25% of total cell media volume) were performed, maintaining 3 mL per dish. Once primary cultures reached ~ 80% confluency, explants were removed and cells were dissociated with 0.25% trypsin–EDTA, 1X (Gibco cat. # 25,200–056; 2.5 g/L trypsin, 0.38 g/L EDTA ≈ 0.91 mM, in HBSS without Ca^2^⁺/Mg^2^⁺, phenol red) until cells detached, and dissociation was stopped by addition of complete L-15 medium containing FBS. The cell suspension was gently triturated, transferred to a sterile tube, and centrifuged to remove residual trypsin; pellets were resuspended in fresh complete medium and seeded into new 60 mm dishes at the indicated split ratios. Across passages, some cell loss during dissociation and transfer was expected, and cultures were reseeded based on the specified split ratios rather than attempting complete mass recovery at each passage. For LtE-1 and LtE-3, the initial subculture (P1) was 1:3 for both lines from explant seeding; subsequent splitting ratios are provided in the morphological and growth assessment section of the methods. For routine maintenance, cultures were maintained in L-15 complete medium with 20% FBS for LtE-1 and 10% FBS for LtE-2 and LtE-3, with ~ 50% medium exchanges three times per week. LtE-1 was sampled across passages 1 to 10 (P1-P10) and P12, while LtE-2 was sampled from P4-P7. The third line (LtE-3) was derived using the same protocol and also maintained for extended passages but was not included in proteomic analyses. All three lines were classified as embryonic-derived lines to indicate that cultures were established at the hatchling/yolk-sac stage (immediately post-hatch), prior to feeding and extensive post-hatch differentiation.

### Morphological and growth assessment of grunion cells

Cultures were inspected daily by inverted phase-contrast microscopy to document morphology and detect contamination. For each passage, time to ~ 80% confluency (t80) was recorded as the number of days from seeding/splitting until cultures reached ~ 80% confluency by visual assessment. Early culture splitting schedules differed by line: LtE-1 was subcultured 1:3 at P1-P2, 1:6 at P3-P5, 1:3 at P6, and 1:6 at P7-P12; LtE-2 was subcultured 1:3 at P1-P2, 1:6 at P3-P4, 1:4 at P5, and 1:6 at P6-P8. The lower splitting ratio at P5 and P6 reflected the transient window of slowed proliferation of the cells between P3-P5. Time-to-80%-confluency (t80) values were recorded at each passage. Population-doubling counts were tracked beginning at the first passage and calculated by dividing the population size of the previous passage at t80 (1 for P1) by the splitting ratio to yield the number of population doublings until the the time the subsequent passage reached t80. Proliferative expansion during the explant outgrowth period was not included due to lack of reliable counts of the number of cells representing the initial outgrowth. LtE-1, LtE-2, and LtE-3 were sustained for extended passages, reaching at least P33 for LtE-1, P10 for LtE-2, and P35 for LtE-3. Phase-contrast micrographs were acquired on a Leica DMi1 inverted microscope using a 10 × objective and a Leica MC120 HD camera, with constant exposure settings across passages. Fiji/ImageJ v1.54p was used for contrast adjustments and addition of scale bars to micrographs. Cultures were monitored routinely for contamination by microscopy and routinely tested negative for mycoplasma (MycoAlert Mycoplasma Detection Kits, Lonza).

### Protein extraction, in-solution trypsin digestion and LC–MS/MS of proteomics samples

Cells were harvested from a single 60 mm dish at > 80% confluency. Two to six replicates (separate 60 mm dishes) were analyzed per passage. For each passage, proteomics replicates reflect independently cultured dishes harvested from the same serially passaged line at that passage (within-derivation replicates), whereas LtE-1 and LtE-2 represent independent derivations initiated from different hatchlings. Cells were rinsed three times with PBS before adding 100 µL lysis buffer (8 M urea, 50 mM ammonium bicarbonate) and scraping them off the plate into lysis buffer using a disposable cell lifter. The samples were then transferred to 0.5 mL protein LoBind microcentrifuge tubes (Eppendorf). Subsequent protein extraction, protein assay, reduction, alkylation, digestion with immobilized trypsin, C18 peptide column cleanup, and peptide assay followed a previously published protocol [85]. Tryptic peptides were buffer-exchanged into 0.1% formic acid in mass spectrometry-grade water using a speedvac (ThermoFisher Scientific Integrated Speedvac; Model #SPD1030-115) and analyzed by online data-independent acquisition (DIA) liquid chromatography-tandem mass spectrometry (LC–MS/MS) with nanoElute2 (Bruker) and Impact II UHR-QTOF (Bruker) instruments as previously described [85]. A 25 cm × 1.5 µm × 150 µm Pepsep C18 column (Bruker) and a 45 min 3–30% acetonitrile gradient were used for online peptide separation prior to nano-electrospray ionization.

### Quantitative analysis and data processing

Raw DIA-MS data were converted to mass spectrometry markup language (mzML) format using msConvert (ProteoWizard) and processed in DIA-NN v2.2.0 using a theoretical spectral library generated from the grunion proteome (*L. tenuis* FASTA, PRJNA1238911 downloaded on May 28, 2025 from NCBI) [86, 87]. Precursor and fragment ion tolerances were set to 0 (default) and automatically determined by DIA-NN (between 10 and 20 parts per million for all samples). Protein false discovery rate (FDR) was controlled at 1%. Common contaminants, cysteine carbamidomethylation, methionine oxidation, and protein N-terminal acetylation were checked for DIA-NN searches and quantitation. The spectral library generated from experimental DIA data by DIA-NN and mzML files were imported into Skyline v25.1 for validation, removal of interferences, removal of non-unique peptides, removal of proteins represented by less than 2 unique peptides and peak refinement with mProphet. Quantitative normalization was performed against the overall sample median and data exported from Skyline for interrogation of transition intensities for peptides into corresponding protein quantities by DirectLFQ v0.3.2 [88]. Morpheus (Broad Institute) was used to assemble per-passage log2 fold-change matrices, sort and rank proteins, and export accession lists for upload to STRING. Passage-specific comparisons of relative protein quantities were performed with two-tailed Student’s t-tests (*p* < 0.05). Enrichment analysis was performed in STRING v12.0 with “proteins with values/ranks” input and FDR set to 1% to obtain STRING functional ‘terms’, referring to individual enrichment entries (GO, Reactome, or KEGG annotations) returned by STRING. Figures were generated in Excel, Morpheus (Broad Institute), and STRING. For STRING Markov clustering (MCL) networks, proteins were filtered for recurrent differential abundance using fixed thresholds; only those that fit significance (up FC > 2; down FC < 0.5) thresholds were included for LtE-1 P1-P3 vs P4 and P5-P7 vs P4. Lists were analyzed in STRING v12.0 with interaction score ≥ 0.400 and MCL inflation = 3. Cluster labels and enrichment terms are reported directly from STRING; upregulated/downregulated counts per passage are derived from fold-change data (FC). Term-count bar charts report the number of STRING functional terms called enriched or depleted at FDR = 1% for each passage vs P4.

## Supplementary Information.

Additional file 1: Table S1. Passage-resolved growth kinetics for LtE lines, including time to ~80% confluencyand cumulative population doublings, with values used to generate growth curves.

Additional file 2: Table S2. Epithelial- and fibroblast-associated marker protein abundances across LtE-1 passages.

Additional file 3: Table S3. Full functional enrichment term listsfor passage comparisons.

Additional file 4: Table 4. Full term lists and scores for LtE-1 longitudinal comparisons versus P4, including pathway/term identifiers used for trendline grouping.

Additional file 5: Figure S1. LtE-1 longitudinal comparison series versus P1 baseline.

Additional file 6: Table S6. Category-aggregate summaries across LtE-2 passages, including stage-level aggregation values referenced in the text.

Additional file 7: Table S7. Unassigned proteins and clusters without a primary STRING description for the Figure 6 subnetworks.

Additional file 8: Table S5. Definitions for letter-coded pathways used in Figure 5 trendline graphs.

## Data Availability

Raw mass-spectrometry files and processed Skyline materials are available on Panoroma Public at https://panoramaweb.org/MMC01KL.url [89]. The protein sequence database (FASTA) used for searching/quantitation was derived from NCBI BioProject PRJNA1238911 (Leuresthes tenuis) [87]. Interactive STRING views corresponding to Fig. 6 are cited in the reference list as Refs [90] and [91].

## Abbreviations

AKT: Protein kinase B
APC/C: Anaphase-promoting complex/cyclosome
C18: Octadecyl (C18) reversed-phase chromatography
DIA: Data-independent acquisition
DIA-NN: DIA-NN software for DIA analysis (DIA-Neural Networks)
DNA: Deoxyribonucleic acid
ECM: Extracellular matrix
EDTA: Ethylenediaminetetraacetic acid
ER: Endoplasmic reticulum
ERAD: Endoplasmic reticulum-associated degradation
ETC: Electron transport chain
FASTA: FASTA-format protein sequence database
FBS: Fetal bovine serum
FC: Fold change
FDR: False discovery rate
GO: Gene Ontology
HBSS: Hanks’ balanced salt solution
KEGG: Kyoto Encyclopedia of Genes and Genomes
LC–MS/MS: Liquid chromatography–tandem mass spectrometry
LtE-1: *Leuresthes tenuis* embryonic-derived cell line 1
LtE-2: *Leuresthes tenuis *embryonic-derived cell line 2
LtE-3: *Leuresthes tenuis* embryonic-derived cell line 3
MAPK: Mitogen-activated protein kinase
MCL: Markov clustering
mRNA: Messenger RNA
MS: Mass spectrometry
mzML: Mass spectrometry markup language format
NCBI: National Center for Biotechnology Information
NF-κB: Nuclear factor kappa B
NMD: Nonsense-mediated decay
PD: Population doubling(s)
PPARα: Peroxisome proliferator-activated receptor alpha
PBS: Phosphate-buffered saline
QC: Quality control
RNA: Ribonucleic acid
rRNA: Ribosomal RNA
SA-β-Gal: Senescence-associated β-galactosidase
STRING: Search Tool for the Retrieval of Interacting Genes/Proteins
TAZ: Transcriptional co-activator with PDZ-binding motif
TCA: Tricarboxylic acid (cycle)
t80: Time to ~ 80% confluency
UHR-QTOF: Ultra-high-resolution quadrupole time-of-flight
WNT: Wingless/Integrated signaling
YAP: Yes-associated protein
