# Supplementary material for: Proteome dynamics during establishment of California grunion (Leuresthes tenuis) cell lines
Source: BMC Biol. 2026 Mar 25;24:108. doi: 10.1186/s12915-026-02577-9 (PMC13137714; doi:10.1186/s12915-026-02577-9)

**Figure S1:** Longitudinal enrichment trends in LtE-1 relative to P1**.**  (A) Reactome bar charts show the accumulation of enriched pathways across passages compared with P1. (B) KEGG pie charts illustrate shifting proportions of functional groups over time. (C-F) Trendline graphs of major categories indicate early elevation of protein turnover and protein synthesis, followed by more stable enrichment of mRNA processing and mitochondrial function in later passages. Definitions of letter-coded pathways used in trendline graphs are provided in Supplementary Table S6.


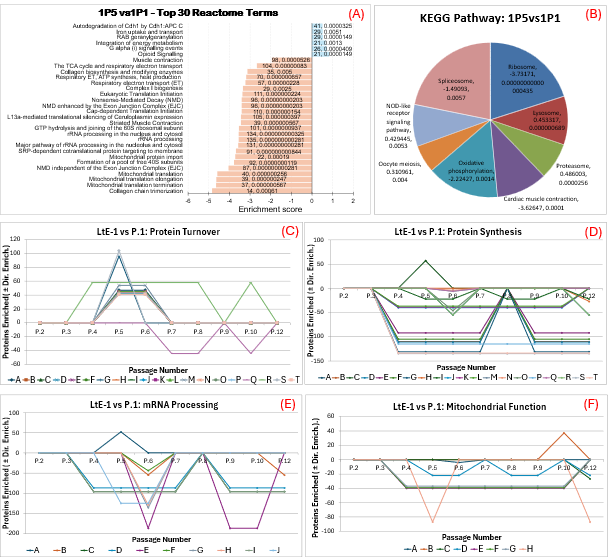

Supplement: Supplementary file 5 — Additional file 5: Figure S1. LtE-1 longitudinal comparison series versus P1 baseline. [file 12915_2026_2577_MOESM5_ESM.docx]
